# Foreign Summary

**Published:** 1839-07-27

**Authors:** 


### FOREIGN SUMMARY.

*Clinical Lecture on Chronic Rheumatism.* By Robert Carswell, M.D.—Gentlemen: I have, to-day, to lay before you an account of cases which have terminated fatally since we last met, both of which present themselves to our consideration in several important points of view. The first was a case of chronic rheumatism, that of Anne King, which I laid before you on a former occasion, and which has afforded us an interesting example of that mode in which rheumatism sometimes terminates fatally, viz., by metastasis to an important internal organ.

The second case to which I have to request your attention, and which I also laid before you on a former occasion, is that of James Shirley, than which it would be difficult to meet with a more striking example of what has been denominated *acute* phthisis, the disease having passed through its successive stages in a period of about four months from the first occurrence of the cough, and in about two months and a half after the first appearance of hæmoptysis; and that, too, in a man who stated that he never had a day's illness before the commencement of the attack which compelled him to seek for relief in this hospital. And not only is this case interesting as regards the rapidity of its progress, but it is equally so from its occurring in a man of great muscular power, having a well-formed and capacious chest, and with no apparent hereditary disposition to the disease. It may, therefore, also be considered as strikingly illustrative of that form of tubercular phthisis denominated *acquired*, in contradistinction to that which is *hereditary*, or transmitted from parents to their offspring. But let us advert to the first case, that of Anne King, ætat 45, who was admitted on the 18th December.

I shall first notice the principal facts of the case, formerly stated to you in detail, and afterwards the most important of those which have since occurred. About two years ago this patient, after getting wet, had rheumatism of the hands and knees, since which time to her admission she never had been altogether free from it. On the contrary, it had been gradually aggravated during the previous eight months. When admitted she complained of gnawing pain in the hands and knees, greatly increased by pressure; the hands were swollen; the fingers flexed, and not extensible; effusion in the knee-joints, especially in the left knee, and both legs flexed and incapable of extension. The pains were somewhat relieved by heat, and increased by cold. There was hot and dry skin; a white but moist tongue; the pulse 80, and regular. The patient was lean and much debilitated.

From this general statement of the case you will perceive that it was one of chronic rheumatism, and of the asthenic character, cold rheumatism, or arthrodynia, the joints being the parts principally affected; the pain being rather relieved than otherwise by heat; and there being little or no febrile excitement.

The treatment adopted consisted in the internal administration of the iodide of potassium, and which, in the course of about a fortnight, the patient then taking a drachm of the solution daily, was followed by considerable improvement, which continued for two weeks longer. At this time, however, the pains became worse, varying much with the state of the weather. She then took the ammoniated tincture of guaiacum, and had sinapisms frequently applied to the knees. This treatment seemed to benefit her for a short time only; the pains again became worse, her general health was less satisfactory, her appetite failed, and she complained much of weakness. The sulphate of cinchonine was then given, with considerable improvement of her general health and strength, and the pain and swelling of the knees diminished under the constant application of poultices, which afforded her more relief than the sinapisms. An unfavourable change, however, induced us again to return to the use of the iodide of potassium, but at the end of a week it was omitted, in consequence of the supervention of febrile symptoms, viz., a quick, hard pulse, considerable heat of skin, sickness, the pains in the knees, too, having considerably increased. These febrile symptoms, however, subsided in the course of two or three days, and permitted us to resume the iodide of potassium, which was again followed by considerable improvement in the space of three days. She complained of little pain in the knees, except when pressed, and they were considerably diminished in size. Her general health, too, was also much improved, and she expressed herself as feeling altogether much better. On the following day, however, a remarkable change for the worse was observed to have taken place. This was on the 3d of March, about two months and a half after her admission. On that day she awoke out of a profound sleep unconscious of where she was, and with no recollection of persons about her. From this state she recovered towards evening, but became delirious during the night. In the morning of the following day the countenance was livid; there was constant muttering and defective articulation; great heat and dryness of the skin; pulse 155, small and weak. The head was not hotter than the other parts of the body. She had no knowledge of persons, and was constantly picking the bed-clothes. Here, indeed, was a remarkable change in the state of the patient, and in the character of the disease, in the space of little more than a day. Marked symptoms of phrenitis had supervened during this space of time, and at a period, too, you will observe, when the local affection had undergone considerable improvement. The patient, as I have said, complained of much less pain in the knees, nor was the pain nearly so much increased by pressure as on the preceding days. The bulk of the knees had also considerably diminished, and the general health improved, up to the day preceding that on which the head affection supervened. Such a sudden and marked change, coupled with the previous amelioration of the chronic local affection, and without any obvious exciting cause, naturally suggested the idea of the occurrence of metastasis, of rheumatic phrenitis, or rheumatic inflammation of the membranes of the brain.

Means were immediately adopted to combat this new and formidable affection. The head was shaved, cold lotions applied to the scalp, and a blister to the nape of the neck. On the following day, and acting on the principle of the disease being of a metastatic nature, blisters were ordered to the knees, or the primary seat of the disease, with the view of exciting anew, or increasing the inflammation in these parts; and to co-operate with this means, and increase its effect, sinapisms were also ordered to be applied to the legs, with the administration of the terebinthinate enema. No benefit, however, followed the use of these measures. The patient died the next day, the fourth day after the commencement of the attack.

I have said that the absence of any exciting cause of the phrenitis in this case, along with the disappearance, as it were, of the rheumatic affection of the knees, were considered by us as sufficient grounds for regarding metastasis to have occurred. Nor does there appear any reason since for supposing that this opinion was ill-founded. The occurrence of metastasis is far from being uncommon (whatever meaning may be attached to this term;) more so, certainly, in gout than in rheumatism; but of the latter numerous cases are recorded of its occurrence in various organs, especially in the heart, stomach, and brain, and of its terminating fatally in persons debilitated by the primary chronic affection alone, or in connection with other deteriorated conditions of the body.

I must not omit, however, to state to you, that our patient seems to have been exposed to a strong predisposing, if not exciting cause of the cerebral affection under which she sank so rapidly. We have been informed that on one or more occasions previous to her attack, she was visited by some itinerant methodist preachers, who had been admitted at the desire of another patient in the same ward; and afterwards a visible alteration was observed to have taken place in her manners, feelings, and sentiments. During her delirium, or on recovering from this state, she expressed herself in language indicative of mental suffering and anxiety, and something like fearful forbodings of punishment for sins with the commission of which she had probably been accused by these too often uncharitable, and not always judicious, visitors of the sick poor of hospitals.

But, however this may be, such was the state of mind of the patient after the interview to which I have alluded, and it is easy to conceive how much the organ thus affected must have been disposed to participate in the morbid condition so long localised in other parts of the body.

The morbid appearances found after death were extremely interesting, as affording us positive evidence of the nature, not only of the chronic disease,—the rheumatism,—from which the patient had so long suffered, but also of that which was the immediate cause of death. Independently of a certain degree of general congestion of the brain, as seen by a greater quantity of blood than usual issuing from the divided vessels in the substance of the brain, together with œdema of the same, and the effusion of serosity into the ventricles; independently of these morbid appearances, I say, there was marked inflammation of the membranes of the brain, more particularly on the *upper portions* of the hemispheres. This consisted in rose-red patches of various sizes, formed by the accumulation of blood in the minute arterial divisions, constituting the capillary congestion of acute inflammation. There was no lymph or pus effused; no opacity of the membranes; hardly any softening of these, and no perceptible alteration of the cortical substance of the brain, contiguous to them. The inflammatory appearances were, in fact, confined to the membranes. It was a case of *phrenitis*, in the strictest sense of the term, and, moreover, of *acute* phrenitis, terminating fatally in the active or early stage; and it destroyed life in this patient the more readily and rapidly that she possessed little bodily energy; her constitution generally, a small amount of reaction; and that the effected organ, in particular, was already in a state of exhaustion from the cause to which I have alluded, before it was submitted to the morbid stimulus of the disease under the influence of which it was deprived of its vitality. And here I may remark, whilst I trust you will not accuse me of indulging in fanciful hypothesis, that the situations of the brain in which the inflammation was most marked, where those in which phrenologists have placed those faculties of the mind which we call *veneration*, *hope*, and *conscientiousness*. Whether these faculties were highly developed or not in this patient I know not, nor could I, had I examined the cranium, have satisfied myself on this point, having neither a theoretical nor practical knowledge of phrenology. However, the fact is as I have stated it. The pathological condition of the membranes of the brain was most conspicuous in the situations which I have named, and if the leading doctrines of phrenology be well founded, it would neither be irrational nor unphilosophical to admit the influence of the moral agency which was exercised on the mind of this patient, and more especially on those individual portions or faculties of it which direct us in the discharge of our religious and moral obligations; and hence it would follow that these having been called into a state of inordinate activity, must, according to the laws of morbid as well as healthy action, have passed into an opposite state, a state of languor or exhaustion, whence might be explained the intellectual derangement and despondency which characterized the cerebral affection in this case.

But I shall not trespass further on your time on a subject of this nature, and concerning which I know so little. I have mentioned it in connection with the anatomico-pathological facts of the case, and the probable origin of the cause in which the disease seemed to have originated.

We now come to a more positive, and therefore more satisfactory part of the case, viz., the diseased appearances found in the principal seat of the rheumatic affection, the knee-joints. They corresponded, in all respects, with those alterations of the joints which, on several occasions, I have had an opportunity of observing after repeated and long-continued attacks of the rheumatism. The cartilages of both knee-joints were extensively destroyed, the articular surfaces of the bones consequently denuded, and in some parts united to each other, or anchylosed. The surfaces in this state were more or less spongy, softened, of a bright-red colour, and in contact with a false cellulo-vascular membrane, of the same red colour, attached to the patella or anterior surface of the joint. The union between certain portions of the surface of the joint, and where the cartilages were destroyed, was pretty intimate, and was broken when the knee was bent after laying open the articular cavity.

All the appearances were those commonly met with in subacute chronic inflammation of long duration, and were such as afforded a satisfactory explanation of the obstinacy of the case, and the partial benefit obtained by the means employed either locally or generally. It is probable, had the patient lived, that the disease would have terminated in complete and permanent anchylosis of the joints.

I believe that none other of the joints that were affected with the disease were examined; nor were there any other morbid appearances observed bearing any relation to the history of the case.

—

*Clinical Lecture*, (*second*,) *on Pulmonary Consumption.*—I have already noticed, generally, the interesting character of the second case which I

laid before you on a former occasion. The more important circumstances which I then related, together with the principal phenomena of the disease up to its fatal termination, I shall now recall to your recollection, and enumerate. This patient, James Shirley, æt. 36, entered the hospital the 30th of January. Although he had enjoyed good health up to a period of two months and a half before his admission, the disease had already arrived at the commencement of the second stage; that is to say, the commencement of softening of the tuberculous matter, and the formation of tubercular excavations, had already taken place.

Cough, from exposure to cold, was the first symptom of the disease, and this was followed by frequent and repeated attacks of hæmoptysis, at short intervals of time, and considerable loss of flesh and strength.

At the time of his admission the cough was severe, accompanied by copious expectoration of a thick, greenish-yellow-coloured tenacious mucus, sometimes tinged with blood. Sonorous and sibilant rattles were heard in various parts of the chest, indicating, along with the characters of the sputa, extensive bronchitis. There was dulness on percussion over the clavicle, on the right side, and posteriorly on the opposite part of the chest, and also a gurgling sound, and occasionally a cavernous ring, or coughing, under the clavicle on the same side,—signs which left no doubt as to the existence of phthisis, of the presence of tubercles, producing by their aggregation the dulness; softening of these, and the commencement of excavations, giving rise to the gurgling sound and cavernous ring.

Besides these physical sounds of phthisis, the usual febrile and hectic symptoms, there was also considerable roughness of the voice, indicating that the larynx was already also implicated in the disease. Under such circumstances no rational hope could be entertained of effecting any permanent benefit for the patient; and, as I remarked to you in my former lecture, the prognosis was, in the highest degree, unfavourable, not only from the physical signs of the disease which we detected at the time the patient was admitted, but from the rapid progress of the case up to this period, and more especially from the severity of the hæmoptysis, which, I said, was generally in proportion to the extent of the tubercular deposition.

Some relief of the cough was obtained by cupping and anodyne expectorants, together with the rest obtained in the hospital, quiet, and a regulated diet. During the second week he was put upon the use of the solution of tartarised antimony, in emetic doses, morning and evening; but, as no amendment appeared to follow its use, and as the patient complained of great fatigue and weakness from its operation, it was laid aside, and the previous treatment resumed. The expectoration, all along very abundant, had now become purulent, or puriform, and offensive; weakness advanced progressively with the copious night perspirations, and although the physical signs were not made the subject of farther investigation by myself, it was obvious that the pulmonary affection was becoming more and more aggravated and extensive. The last few nights before the death of the patient there was more or less delirium, and on the day also on which he died, we found him, unexpectedly, much agitated, moving about in bed, and talking incoherently,—with great dyspnœa, extreme congestion of the face and lips, the hands and feet; with coldness of the extremities, and the pulse at the wrist almost imperceptible.

The rather sudden and unexpected occurrence of these symptoms we attributed to an attack, most probably, of pneumonia, or pleuro-pneumonia, as the post-mortem appearances proved to have been the case. The state of the lungs will show you to what an extent the tubercular deposition may take place in a short period of time, and, when complicated with bronchitis, prove fatal before the tissue of the lung is much destroyed, or before the tuberculous matter has undergone extensive softening.

Before pointing out to you the morbid appearances found in the lungs, I may state that there was effusion into the cavity of the pleura and pericardium, and which always happens in cases similar to the present, in consequence of the pulmonary congestion, and which necessarily contributes to the increase of that state, and to hasten death by asphyxia. In consequence, also, of this state of the pulmonary circulation, together with the mechanical obstruction offered by the immense quantity of tuberculous matter contained in the lungs, the right side of the heart was distended with coagula of fibrinous blood, whilst the left contained a much less quantity of coagulated blood only, of a very dark colour. This difference was worthy of notice, as indicating not only the mode of death by asphyxia, from obstructed pulmonary circulation, but also the almost complete cessation of the special function of the lungs some time before death, the blood appearing to have passed through these organs to the left side of the heart, without having undergone the physiological change from the venous to the arterial state,—a circumstance which must also have contributed to hasten the period of dissolution. The congestion was also observed in the other organs, as the liver, spleen (which was very large and soft,) kidneys, and, in some degree, in the stomach.

The presence of tubercles was not confined to the lungs,—they were found in the intestines, the organs in which, *in adults*, they are most frequently met with after the lungs; or if tubercles are not found, the consequences of them are, viz., more or less ulceration. In this case we had both, but chiefly tubercles, and these were confined to the follicular structure of the intestines, the isolated and agglomerated follicles, and will afford you a beautiful illustration not only of the primary seat of the tuberculous matter in the intestines, but of the extensive ulcerations to which they give rise in the last, or colliquative stage of phthisis. We have also an instructive example of the morbid condition of the larynx, produced by the same cause as the ulcerations in the intestines, which occasions the roughness of the voice and aphóny which takes place in the phthisis. These appearances I shall now endeavour to point out to you.

Those of you who were present at the post-mortem will remember that the lungs were seen greatly enlarged, and did not collapse on laying open the cavity of the chest; circumstances which you will find satisfactorily explained by the presence of the immense quantity of tuberculous matter which these organs contained, and the state of inflammatory congestion, hepatisation, and œdema, which they presented.

On raising the lungs, cellular adhesions were seen uniting the pleuræ superiorly and posteriorly on the left side, and superiorly on the right side.

The left lung, as you perceive, presents externally a mottled aspect of red, purple, gray, and yellow, produced by the accumulation of arterial and venous blood in the parenchyma of the lungs and subpleural cellular tissue, and masses of tuberculous matter of various sizes; it feels very heavy, and almost as solid as liver. When laid open by a vertical section from the summit to the base, you perceive that it is chiefly composed of tubercles, grouped into irregular rounded masses varying from the size of hemp-seed to that of one or both fists. The tuberculous matter is readily recognised by its pale-yellow colour, opacity, and cheesy aspect and consistence,—the physical character which it usually presents in the crude state, as it is called. The softening of this matter, together with the destruction of the pulmonary tissue, have taken place in three separate portions of the lung, and the result has been the formation of what are called tubercular excavations. Two of these, of moderate size, occupy the summit of the lung, and a larger cavity is seen lower down and towards the outer side of the upper lobe, immediately beneath the pleura, the surface of which is covered by an organised false membrane of considerable density, which may, possibly, have prevented the occurrence of perforation of the pleura. The tubercular excavations present an irregular form, are but slightly anfractuous, and contain a quantity of muco-puriform matter, the secretion of the adventitious mucous membrane with which they are lined.

The small quantity of pulmonary tissue which remains unoccupied by tuberculous matter presents the appearance observed in the first and second stages of pneumonia, viz., an accumulation of blood, with some œdema; a certain degree of crepitation still remaining on pressure in some parts; and a still greater accumulation of blood, complete absence of crepitation, and diminished cohesion of the pulmonary tissue, in other parts. This lung weighs four pounds and a half. The right lung also contains a great quantity of tuberculous matter, which is much more abundant in the upper than in the other lobes, and is in the same stage as in the left lung. The upper lobe contains two excavations similar to those already described. The pulmonary tissue is generally congested and œdematous, but crepitant. The weight of the lung is three pounds thirteen ounces.

The mucous membrance of the larynx is somewhat swollen, but pale; that of the trachea presents a multitude of circular, superficial ulcerations, which, being nearly of the same colour as the mucous membrane, which was slightly reddened, might have been overlooked by an inattentive observer. They are best seen by holding the trachea in an oblique position, as a shadow is then thrown inwards from their edges, and brings them into relief. They are, also, by far, more numerous on the right than on the left side of the trachea, a circumstance worthy of your attention, as it corroborates the opinion of Louis, that they arise from the long-continued irritation produced by the passage or contact of the morbid secretions of tubercular excavations and inflamed bronchi, favoured by the *position* of the patient. In this case the patient lay almost continually on the *right side*, and, as you have seen, the ulcerations were greatly more numerous on *this side* of the trachea, where, consequently, the influence of the exciting cause must have been more extensively and more frequently in operation than on the opposite side. The hoarseness, and, occasionally, partial aphony which occurred, particularly during the latter stages of the disease, were the consequence of these morbid states of the larynx and of the trachea.

With regard to the intestines, we find in them the same morbid alterations which we have just seen to exist in the trachea, as consequences of the tubercular disease. But, as most frequently happens in cases similar to the present, I mean in acute phthisis, these alterations are neither so extensive nor so conspicuous, as in chronic cases of this affection. They are not, however, on this account, less instructive in this instance, for we have them here, in several portions of the small intestine, in the early stage. Ulceration, so very common, and frequently so very extensive, in most cases of chronic phthisis, is seen only commencing in a few points of the ileum, and where the follicles had previously been the seat of tubercular deposition. That the tubercular deposition preceded, and was the cause of the ulceration here, as it preceded and was the cause of the excavations in the lungs, may be admitted from the state of the follicular structure in other parts of the intestine. In the glands of Peyer, or the glandulæ agminatæ; and also in some of the glandula solitariæ, the tuberculous matter is distinctly seen accumulated in rounded masses varying from the size of a pin's head to that of hemp-seed, the surrounding mucous membrane being either slightly injected with red vessels or of its natural colour. In contiguous portions the mucous membrane is partly ulcerated, and somewhat congested, or quite pale. The ulcers are small, round, and smooth, and appear as if they were the result of the separation or discharge of the tuberculous matter from distended or partially destroyed follicles. These appearances are observed principally in the inferior portion of the ileum, as happens in the great majority of cases of phthisis. But they are also seen in the caput cœcum coli. The very limited extent of the ulceration, and the very slight traces of the existence of inflammation in the intestines, enable us to account for the absence of colliquative diarrhœa in this case.

I shall not detain you by an attempt to explain the physical signs of the pulmonary affection by the lesions found in the lungs after death, and for this reason, that we did not employ either auscultation or percussion for a considerable time before the death of the patient. There is no doubt as to the local disease having made very great progress after the admission of the patient; that a great part of the tubercular deposition in the left lung took place after this period, and that the supervention of pneumonia of this lung was the immediate cause of death. The disease in the right lung was, as the physical signs clearly demonstrated, in a more advanced stage at, and some time after, the admission of the patient, than it was in the left lung; although the post-mortem examination has shown us that at the fatal termination of the disease, the latter lung was more extensively affected than the former. That this difference was not known during life is only attributable to the circumstance already noticed, viz., our not having examined the chest for a considerable time previously, from a desire not to disturb the patient, the nature and issue of whose case was but too obvious.—*Lancet.*

---

*Relative Frequency of Phthisis in different Climates.*

A proposition was made two or three years since, in the French Academy of Medicine, for the purpose of obtaining correct information as to the relative frequency of phthisis in different climates. This was to be obtained by the aid of these correspondents scattered through different parts of the world. There are many difficulties in the way of this arrangement, and it will, perhaps, never produce the good effects which were anticipated from it. A more feasible means of arriving at the truth is afforded by the British army surgeons, who are enabled to observe the relative frequency of development of phthisis amongst healthy individuals, placed nearly in the same condition as to exercise and food. From the reports of the army surgeons, we extract the following interesting statement, which we copy from the Dublin Journal, not having at hand the original document. It will be seen that the colder regions of our continent are by no means so favourable to the development of tuberculous diseases of the chest, as the milder temperature of southern stations. There is, however, one cause of error, which should be recollected. That is, the troops who are sent from Great Britain to tropical climates, are removed to a climate much more unlike their own, than those who are stationed in Canada; hence it is probable that the entire change of constitution which follows, may be, to a great degree, productive of phthisis in those who are disposed to tuberculous affections.

There is one undoubted inference, that is, individuals labouring under phthisis may, perhaps, be benefited by a short stay of a few months in a tropical climate, but are very probably injured by a protracted residence. The effects of this residence are shown by the fact, that of a thousand troops, there are annually attacked with consumption in the United Kingdom 6.6, in Gibraltar 8.2, in Malta 6.7, in the Ionian Islands 5.3. That is, the disease is more fatal in the Mediterranean stations, (with one exception,) than in the British Islands. It is already well known that the same is true of the West Indies:

"*Deductions from the Report.*—Having, in the course of this report, frequently adverted to the uniform degree of prevalence which, notwithstanding the dissimilarity of climate, has been found to exist in the proportion of pulmonary affections in Nova Scotia and Canada, compared with Malta and Gibraltar, it seems unnecessary here to recapitulate the evidence on that subject; but it may be proper to inquire whether there exists, in the moral or physical condition of the troops in the Mediterranean and American stations, any difference likely to have influenced the results on which that comparison has been founded.

In the last section of the West India Report we showed that these diseases, even under the high temperature of the tropics, prevailed to a greater extent than in the United Kingdom. But it may be argued that several circumstances, independent of climate, were there in operation to induce that peculiarity; for instance, the innutritive qualities of the diet, the limited and defective state of the barrack accommodation, and the general prevalence of intemperance, were all causes tending to affect the health of the troops in no inconsiderable degree. We are, therefore, led to inquire whether any deteriorating circumstances of a similar nature exist in the Mediterranean from which the troops in North America are exempt, and by which the tendency to these diseases may have been so far aggravated as to counterbalance the advantages otherwise resulting from its mild and equable climate.

So far, however, from this being the case, every circumstance has been more favourable to the troops in the Mediterranean. The barrack and hospital accommodation, in Malta and Gibraltar at least, is not only of a more substantial nature than in Canada and Nova Scotia, but nearly double the space is allotted to each soldier; the diet, with the exception of a greater issue of salt meat in Gibraltar during the winter months, is nearly the same, and the meals are regulated on similar principles. Intemperance, to which so much has been attributed as an exciting cause of these diseases, cannot be said to prevail to a greater extent in the Mediterranean than America, where the constant use of ardent spirits is likely to prove still more prejudicial than the low wines which form the principal medium of intoxication in the Mediterranean.

In all these respects, then, the troops in the Mediterranean have decidedly the advantage. We have yet to advert to another circumstance no less favourable to them: consumption, the most fatal of this class of diseases, is supposed to affect persons at an early period of life more than those of mature age. Now, owing to the frequency of desertion in North America, so many recruits have to be sent out from this country that nearly one-half of the force there is under twenty-five years of age, while in the Mediterranean, where no such necessity exists for large drafts from the depôts, the proportion under that period of life is only from a third to a fourth of the whole; consequently the composition of the force in the Mediterranean renders it much less subject to the influence of consumption, if not also of the other pulmonary diseases which frequently precede it.

When we find, notwithstanding all these circumstances apparently so favourable to the greater development of these diseases in Canada and Nova Scotia, that the troops there do not suffer from them to a greater extent than in the Mediterranean, it would manifestly be incorrect to attribute their prevalence in North America to the reduced temperature and sudden atmospherical vicissitudes incident to that quarter of the globe, seeing that the sufferings of the troops from these diseases are equally great in other climates where no such causes are in operation to induce them.

We have been thus particular on this head, because, in the reports from the different medical officers in North America, we find a great portion of the sickness and mortality attributed to the severe and changeable nature of the climate inducing pulmonary affections of various kinds. It is true that many of the deaths arise from these causes, but in this respect the troops there are by no means singular in their sufferings, for, throughout the wide extent of the British Colonies, few stations can be found where soldiers are not affected by them in an equal degree, though, perhaps, owing to the greater extent of mortality by other diseases, these are less a subject of observation or remark. In addition to the instances already adduced on that head, it will be shown, in a future report, that even in the mild climate of the Mauritius, more soldiers are attacked by consumption, and nearly as many by inflammation of the lungs, as in the most inclement regions of North America, though we do not find that the prevalence or fatal character of these diseases attracts so much attention."

*Case of the Accidental Administration of forty grains of Extract of Belladonna.* By O. Clayton, Esq.—The author's motive in relating the above-named case, the subject of which recovered from the effects of the poison, was that the order of succession of the symptoms differed from that described by Dr. Christison; for in it sopor preceded the delirium, which did not come on for six hours after the administration of the poison, whereas the reverse is commonly the case. The author considers it also worthy of remark that the pulse, which was one hundred and sixty half an hour after the poison was taken, fell in twenty hours to fifty-eight, and that it varied from one hundred and sixty to one hundred and twenty during the sopor, but did not reach more than ninety-five during the delirium.

Mr. Cæsar Hawkins referred the author of the paper to a case in St. George's hospital, some time since, under Sir B. Brodie, in which the patient swallowed nearly an ounce of the extract of belladonna, and yet recovered.

Dr. J. Johnson considered that as the stomach-pump was applied twenty-five minutes after the swallowing of the poison, nineteen-twentieths were brought up, and consequently no fact was adduced in support of the quantity that could be safely borne. He thought that if the forty grains had been retained, the effect would have been fatal. The largeness of the dose argued nothing, for half an ounce of the powder of ipecacuanha would produce no greater effect than five grains, as the former quantity would be immediately rejected, before any extra-physiological effect could be produced upon the stomach.

*Lancet.*

---

*Scald Head.*—A child who labours under this affection, and who is under Sir B. Brodie's care, had had several different applications made to the shaven scalp, but without any benefit being derived from them. The latter treatment has more particularly consisted in having the head shaved, and the scalp lightly touched with the strong acetic acid. This, however, caused so much pain, that it was necessary to stop it; and thus the remedy has not had a fair trial, nor is the disease in the least degree abated. Sir B. Brodie ordered the head to be again shaved, well washed with brown soap and water, and the ointment of the nitrate of mercury applied to it every morning. Whilst standing by the patient's bedside, Sir B. Brodie inquired whether any other surgeon, either in or out of the hospital, had seen the case, or given their opinion upon it, which question being answered in the negative, he observed that the red precipitate of mercury, in the proportion of two drachms to one ounce of ointment, was an exceedingly valuable preparation in some cases of the kind. He was acquainted with a surgeon who cured such cases by subjecting the scalp to the steam of hot water for one hour daily.

*Ibid.*

